# Choice-induced inter-trial inhibition is modulated by idiosyncratic choice-consistency

**DOI:** 10.1371/journal.pone.0226982

**Published:** 2019-12-26

**Authors:** Christian Wolf, Alexander C. Schütz

**Affiliations:** 1 AG Allgemeine und Biologische Psychologie, Philipps-Universität Marburg, Marburg, Germany; 2 Allgemeine Psychologie, Westfälische Wilhelms-Universität, Münster, Germany; Shandong University of Science and Technology, CHINA

## Abstract

Humans constantly decide among multiple action plans. Carrying out one action usually implies that other plans are suppressed. Here we make use of inter-trial effects to determine whether suppression of non-chosen action plans is due to proactively preparing for upcoming decisions or due to retroactive influences from previous decisions. Participants received rewards for timely and accurate saccades to targets appearing left or right from fixation. Each block interleaved trials with one (single-trial) or two targets (choice-trial). Whereas single-trial rewards were always identical, rewards for the two targets in choice-trials could either be identical (unbiased) or differ (biased) within one block. We analyzed single-trial latencies as a function of idiosyncratic choice-consistency or reward-bias, the previous trial type and whether the same or the other target was selected in the preceding trial. After choice-trials, single-trial responses to the previously non-chosen target were delayed. For biased choices, inter-trial effects were strongest when choices were followed by a single-trial to the non-chosen target. In the unbiased condition, inter-trial effects increased with increasing individual consistency of choice behavior. These findings suggest that the suppression of alternative action plans is not coupled to target selection and motor execution but instead depends on top-down signals like the overall preference of one target over another.

## Introduction

While humans interact with their environment, they constantly choose between multiple possible actions. Effective behavior requires that action plans are selected based on behavioral goals and that non-selected action plans are suppressed. Selection among multiple action plans can be optimized by considering the expected value of options. Such a selection process based on value information is not only determined by top-down factors, but also by the history of reward-based selection [1–5]. Learned reward associations can bias covert [6] as well as overt attentional selection [7] and continue to do so even when they compete with the top-down goals of the momentary task [8]. Biases due to previous selection processes can also operate on shorter timescales, as it is often observed in inter-trial priming [9–12]. Inter-trial priming effects can be evoked by the reward structure of the previous trial [13]. For example, ignoring a distractor can be impaired when this distractor served as a rewarded target in the previous trial [14–16].

Recently, we showed that when a decision is formed based on value information, a response to the non-selected action is delayed in the subsequent trial [17]. In our previous study [17], participants received a small monetary reward for correct saccades to targets appearing left or right from fixation. Targets in the left and right hemifield differed in their reward magnitude. We interleaved trials with one target (single-trials) and trials in which both targets were displayed (choice-trials). In choice-trials participants were free to choose between the two targets, and almost exclusively decided for the target associated with a higher reward. We analyzed the reaction time (latency) in single-trials as a function of reward magnitude and whether choice-trials were interleaved in the same block or not. In blocks without choice-trials, we did not find any evidence for a latency difference between saccades to low and high reward targets. When choice-trials were present, single-trial responses to the less rewarded target were delayed. These results were partly caused by inter-trial effects: After choice-trials, saccades to the less rewarded (i.e. non-chosen) target were delayed.

Why does a decision between two rewarded targets delay subsequent responses to the non-chosen target? One potential explanation is lingering inhibition: In order to successfully select the high rewarded target in choice-trials, a response to the less rewarded target needs to be suppressed. This suppression might survive the inter-trial interval and might slow down a response to the suppressed target. This possibility would predict that the magnitude of inter-trial effects critically depends on the length of the inter-trial interval. Evidence for this comes from a study by Dorris et al [18] who observed strongest sequence effects at short intervals. However, this lingering inhibition hypothesis is not consistent with findings from a recent EEG study. This study investigated the influence of choices on single-target reactions using manual responses. The reaction time pattern [19,20] was similar to the reaction time pattern in the same paradigm using saccades as effector [17]. Moreover, there was a pre-stimulus lateralization of alpha power in blocks with a high proportion of choice-trials [19]. Alpha oscillations are a robust marker of inhibitory control and attentional selection [21]. Lingering inhibition would predict that the lateralization of alpha power is maximal at the beginning of the pre-stimulus interval and decays over time. In contrast to that, alpha lateralization increases during the early pre-stimulus interval [19]. This suggests that choices cause an inhibition of the non-chosen target and that this inhibition builds up at the beginning of a trial rather than being directly inherited from the previous trial. Consistent with this inhibition interpretation, modelling saccade latency distributions using the LATER (Linear Approach to Threshold with Ergodic Rate) model [22] showed that the baseline activation was reduced for single-trials to the less rewarded target [17].

Inhibition due to previous decisions can build up, for example due to retrieval effects. Retrieval effects can be explained in the event-coding framework [23,24]: task context, stimulus and response of a given trial are bound into a common event file and stored in memory. If the same stimulus is encountered in the next trial, these event files are said to be automatically retrieved. Not only task-relevant but also task-irrelevant information can be stored in event files [25,26] and responses can be impaired when encoded and retrieved information do not match [27]. The two choice-trial targets and the response might thus be stored in a common event file. If the non-chosen choice target subsequently appears as a single target, this might activate a response to the opposite hemifield and thus impair the response, leading to the inter-trial effects mentioned above [17]. This would predict that inter-trial effects would occur after a choice-trial no matter whether there is a reward difference between the two choice-trial targets or not. Alternatively, a choice-trial might update the internal preference of one target over the other, leading to stronger proactive preparation to select the high reward target. This hypothesis would predict that inter-trial effects can only be observed when there is a clear preference of one target over the other.

Here, we aimed to dissociate the two possibilities (i) that choices lead to inter-trial inhibition in subsequent single-trials only when there is a reward-bias between both choice targets and participants *proactively* prepare a saccade to the high value target or (ii) that inter-trial effects are a consequence of executing the previous saccade (*retroactive*) and thus also occur without any reward bias. To do so, we compared inter-trial effects in blocks where participants chose between two targets of either a different or the same reward. When both choice targets yield the same reward, there is no external reason to prefer one target over the other, and choice behavior should be more, but not necessarily completely balanced [28] compared to the condition where one target yields a higher reward.

## Materials and methods

### Participants

We recorded data of 32 participants (21 female, 5 left-handed according to self-report) with a mean age of M = 23 years (SD = 5, range = 18–44 years). The number of participants was pre-registered and based on effect sizes of previous findings [17]. All participants were students of Marburg University, had normal or corrected-to-normal vision and signed written informed consent prior to testing. As a compensation, participants received course credit or 8€/h. Additionally, participants received a reward based on their performance. This reward ranged between 4.20€ and 5.50€ (M = 5.20€) and could not be transferred into course credit. The experiment reported in this study was approved by the ethics committee of the Psychology department at Marburg University (proposal number 2017-27k) and conducted in accordance with the declaration of Helsinki.

### Apparatus

Experiments were presented on a VIEWPixx monitor (VPixx Technologies Inc., Saint-Bruno, Quebec, Canada) using MATLAB (The Mathworks, Natick, MA, USA) and the Psychtoolbox [29]. The monitor had a spatial resolution of 1920 × 1080 pixel, a size of 51.5 × 29 cm and was viewed from a distance of 60 cm. Eye movements of the right eye were recorded using a desktop mounted EyeLink 1000 (SR Research Ltd., Ontario, Canada) with a sampling rate of 1000 Hz and the Eyelink Toolbox [30].

### Procedure

**Fig 1:**
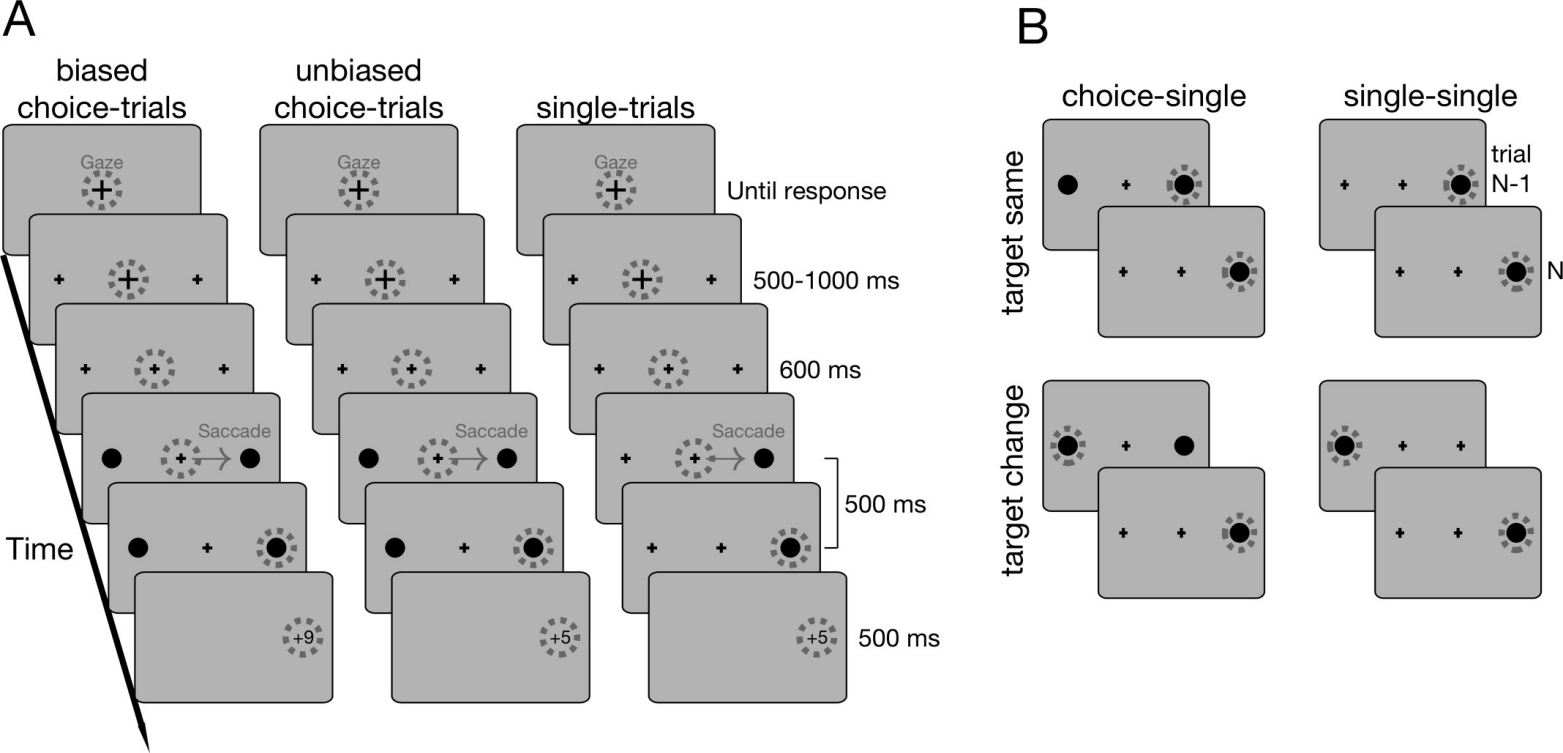
(A) Trial-procedure for biased and unbiased choice-trials as well as for single-trials. Biased and unbiased choice-trials differed with respect to the reward distribution across both target locations. In single-trials, only one target was displayed and reward was the same for both locations. One experimental block contained single-trials and either biased or unbiased choice-trials. (B) Inter-trial sequences. For a given single-trial, the previous trial could be a choice- or single-trial and it could imply a saccade to the same or the other target. To compute inter-trial effects, we subtracted single-trial latencies from choice-single sequences from the corresponding single-single sequence. Sequences were further split into trials from the biased and unbiased condition (not shown in figure). (A & B) Gray dashed circles denote gaze position and thus indicate which target was selected in choice-trials.

At the beginning of a trial, a black fixation cross with a diameter of 0.5° appeared at screen center on a gray background (Fig 1A) and signaled participants to start a trial by pushing the spacebar on a keyboard while maintaining fixation. Two placeholders, crosses with diameters of 0.25°, appeared left and right from fixation at 15° eccentricity. After a random time interval between 500 and 1000 ms, the central fixation cross reduced its size to 0.25° to indicate the onset of the target in 600 ms. Targets were black dots with a radius of 0.25° and were presented for 500 ms. Participants had to maintain fixation until the target appeared and then shift their gaze to the target during its presentation. In successful trials, the presentation of the target was followed by the presentation of the obtained reward and the accumulated score (e.g. “+9 | 42”) at the chosen target location. If participants looked at the placeholder in single-trials or did not make a saccade, they obtained no reward and a “+0” was shown at the end of the trial. Rewards were score points (1, 5 or 9) and participants received 1€ for every 500 points. In single-trials, participants always received a reward of +5. The same holds for choice-trials in the unbiased condition. In the biased condition however, one target/hemifield was assigned a high, “+9”, the other one a low reward, “+1”. At the beginning of each block, participants were informed about the condition (biased or unbiased) and the distribution of reward (e.g. left or right choice-target highly rewarded). Participants completed four blocks, each comprising 120 trials of which 40 were choice-trials and the remaining 80 were single-trials, equally balanced with regard to location. One half of participants started with two blocks of the unbiased condition, the other half with two blocks of the biased condition. In the biased condition, the two blocks differed with regard to which hemifield was highly rewarded. The order of high reward locations was balanced across participants. After the experiment, participants were debriefed and asked for strategies during choice-trials and whether they noticed a preference for one side.

### Analyses

Analyses plans, except for the potential effect of block order and the correlational analyses were pre-registered at the open science framework (doi: 10.17605/OSF.IO/8BFU4). All underlying data are publicly available from zenodo.org (doi: 10.5281/zenodo.1406152). We determined saccades using the EyeLink 1000 algorithm and defined latencies as the first saccade sample with respect to target onset. Targets were labelled as chosen when gaze was within a square region of 2° around the target. We only considered trials with latencies between 95 and 425 ms. Based on this criterion, we discarded 4.5% of trials. In addition to the latency-criterion and in addition to our pre-registered analysis plan, we excluded trials with an amplitude below 11°. This affected further 0.4% of trials. The rationale for this was to remove trials in which the target was foveated with more than one saccade. Removing these trials did not affect the results and conclusions drawn. Discarded trials were analyzed as errors.

To analyze inter-trial effects, we first determined individual choice-preferences in each of the four experimental blocks. Therefore, we specified which target was chosen more frequently in choice-trials. If both targets were chosen equally often within a block, we defined the target with lower choice-trial latency as preferred target (this happened once in the unbiased condition). The overall choice consistency was then determined by averaging the choice consistency across both blocks of the same condition, no matter whether the same or a different target was preferred. In a second step, we determined (i) single-trial latencies which occurred after a choice-trial (choice-single sequence) and (ii) single-trial latencies which occurred after other single-trials (single-single sequence). There were 4 corresponding choice-single and single-single sequences: Each sequence could either imply a change in saccade direction or not (“target change” versus “target same”) and it could belong to the biased or to the unbiased condition (Fig 1B). For every individual in each of the four sequence types, we then subtracted the mean single-single latency from the mean choice-single latency. Inter-trial differences >0 would imply higher single-trial latencies after choices. We only considered sequences with two correct trials. For the biased condition, we only considered choice-single sequences with choice-trials to the highly rewarded target. In this case, a change in target after a choice-trial would always imply a saccade towards the target with less reward in choice-trials and we made sure that this was also true for the corresponding single-single sequence.

Inferential statistics were carried out in MATLAB, R (3.3.2; R Development Core Team, 2016) and JASP version 0.10.2 [31]. Single-trial latencies and inter-trial effects were analyzed using repeated-measures ANOVAs and followed by post-hoc t-tests with Bonferroni-Holm-corrected α-level. For ambiguous or non-significant post-hoc tests, we also report Bayesian paired t-tests using default prior values in JASP. Bayes Factors (BF_10_) >1 favor the alternative, and values <1 favor the null-hypothesis. The more BF-values deviate from 1, the stronger the evidence. Values between 0.33 and 3 are typically considered inconclusive evidence [32]. To analyze single-trial errors, we used a linear mixed model to compare the difference in error rates between preferred and unpreferred targets for the two reward conditions, because error rates were not normally distributed. The model contained fixed effects of reward and preference and random effects of participant.

## Results

**Fig 2:**
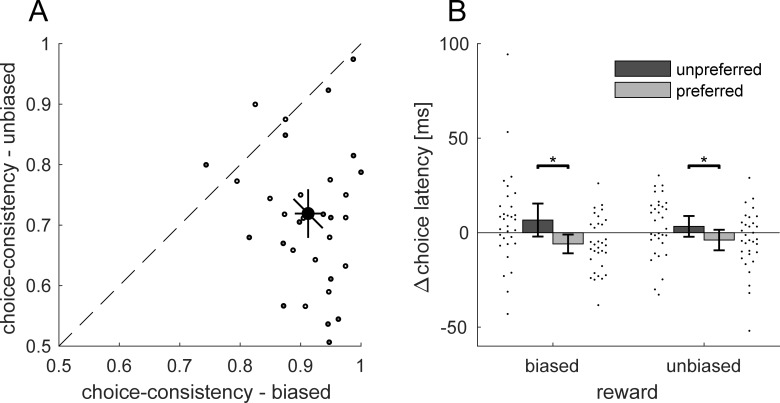
Choice behavior. (A) Choice-consistency expressed as *proportion preferred target chosen* for the biased and the unbiased reward condition. Open and grey-filled circles denote participants who started with the unbiased and biased condition respectively. The black symbol indicates the mean choice-consistency across all participants. The diagonal error bar denotes the variability of the difference between the two measures and has to be compared against the dashed diagonal. (B) Biased and unbiased choice-trial latencies split in preferred (light) and unpreferred (dark) targets. Data are plotted as the difference to the individual mean. All error bars denote 95% confidence intervals. Black data points next to each bar graph denote values of individual participants in that respective condition. Asterisks indicate a significant difference (p < 0.05).

In a first step, we analyzed choice behavior. If our manipulation was successful, then choices should be less consistent in the unbiased compared to the biased condition. In the biased condition, all participants in all blocks more frequently chose the high-reward target (Fig 2A). In the unbiased condition where both choice targets yielded the same reward, 28 out of 32 participants preferred the same target across both blocks. For every block, we computed the proportion with which participants chose their preferred target. Choice-consistency was higher in the biased, M = 0.91 (SD = 0.06), than in the unbiased condition, M = 0.71 (SD = 0.12), as revealed by a Wilcoxon signed-rank test, *z* = 4.64, *p* < 0.001. Choice behavior differed in terms of consistency but not in terms of reaction time: Average choice latencies were M = 234 ms (biased-unpreferred), M = 221 ms (biased-preferred), M = 231 ms (unbiased-unpreferred) and M = 223 ms (unbiased-preferred). Fig 2B shows choice latencies relative to the individual mean. Biased and unbiased choice latencies were not any different, neither for the preferred, *t*(31) = 0.49, *p* = 0.626, *BF*_*10*_ = 0.211, nor for the unpreferred target, *t*(30) = 0.563, *p* = 0.578, *BF*_*10*_ = 0.222.

To exclude effects of block order on choice behavior, we compared choice consistency for participants who started with the biased condition to those participants who started with the unbiased condition. Choice consistency in unbiased choices was not different for participants who first completed the biased (M = 0.71; SD = 0.14) or the unbiased condition (M = 0.72; SD = 0.09), *t*(30) = 0.119, *p* = 0.906, *BF*_*10*_ = 0.338. The same holds for choice latencies to the preferred target in unbiased choices (biased first: M = 224 ms, SD = 28 ms; unbiased first: M = 222 ms, SD = 17 ms), *t*(30) = 0.27, *p* = 0.788, *BF*_*10*_ = 0.346.

**Fig 3:**
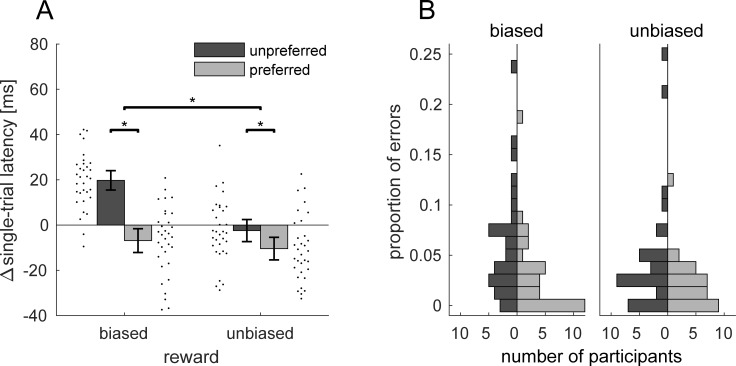
Single-trial behavior. Single-trial latencies (A) and errors (B) for preferred (light) and unpreferred (dark) targets. (A) Single-trial latencies in every condition are expressed as the difference from the individual mean. Black dots next to each bar graph denote values of individual participants in that respective condition. Error bars denote 95% confidence intervals of between-participant variability. Asterisks indicate significant (p < 0.05) comparisons. (B) vertical histogram of the proportion of single-trial errors.

In a second step, we analyzed single-trial behavior. Single-trials were classified as belonging either to the biased or unbiased condition and according to the individual choice preference in that block to the preferred or unpreferred location in choice-trials. We expected higher latencies in the biased condition for the unpreferred compared to preferred targets. This would be a replication of our previous study [17]. These differences were partly caused by choices slowing down responses to the non-selected target in the subsequent trial [17]. A similar pattern for single-trial latencies in the unbiased condition would support the hypothesis that choices exert a retroactive influence. To the contrary, if there was no single-trial latency difference in the unbiased condition between preferred and unpreferred target, this would suggest that choices exert a proactive influence. Average latencies in single-trials were M = 237 ms (SD = 31 ms) for the biased-unpreferred, M = 210 ms (SD = 32 ms) for the biased-preferred, M = 215 ms (SD = 27 ms) for the unbiased-unpreferred and M = 207 ms (SD = 26 ms) for the unbiased-preferred condition. Fig 3A shows single-trial latencies as ipsative data (i.e. the difference of every data point from the individual mean). We analyzed single-trial latencies using a 2×2 repeated-measures ANOVA with the factors reward-bias (biased versus unbiased) and target (preferred versus unpreferred target). On average, latencies for the unpreferred target were higher, main effect of preference, *F*(1,31) = 80.2, *p* < 0.001, *η*_*p*_^2^ = 0.72. This was true for both levels of reward-bias, the biased, *t*(31) = 11.7, *p* < 0.001, *d* = 2.06 and the unbiased condition, *t*(31) = 3.09, *p* = 0.004, *d* = 0.55. Moreover, we observed a main effect of reward-bias, *F*(1,31) = 8.9, *p* = 0.006, *η*_*p*_^2^ = 0.22. Latencies were on average higher in the biased condition (M = 224 ms) compared to the unbiased condition (M = 211 ms). Additionally, the ANOVA also revealed a reward-bias × preference interaction, *F*(1,31) = 39.4, *p* < 0.001, *η*_*p*_^2^ = 0.56. Latencies from biased and unbiased condition only differed for the unpreferred location, *t*(31) = 5.21, *p* < 0.001, *d* = 0.92, but not for the preferred location, *t*(31) = 0.73, *p* = 0.469, *d* = 0.13, *BF*_*10*_ = 0.24.

It is possible that these latency differences simply reflect a speed-accuracy trade-off. If this was the case, then participants should be more accurate in their responses to the unpreferred target. To this end we analyzed erroneous single-trials, thus, single-trials in which a saccade response was made too early, too late, towards the placeholder in the other hemifield or where the amplitude was below 11 deg (saccade gain < 0.73). Latency results cannot be explained by a speed-accuracy trade-off, because single-trial error probability (Fig 3B) was higher for the unpreferred (biased: M = 0.06, unbiased: M = 0.04), compared to the preferred target (biased: M = 0.03, unbiased: M = 0.03), *F*(1,31) = 15.84, *p* < 0.001. Biased/unpreferred errors were mostly due to too late saccades (40%) or trials without responses or responses to the placeholder (37%).

**Fig 4:**
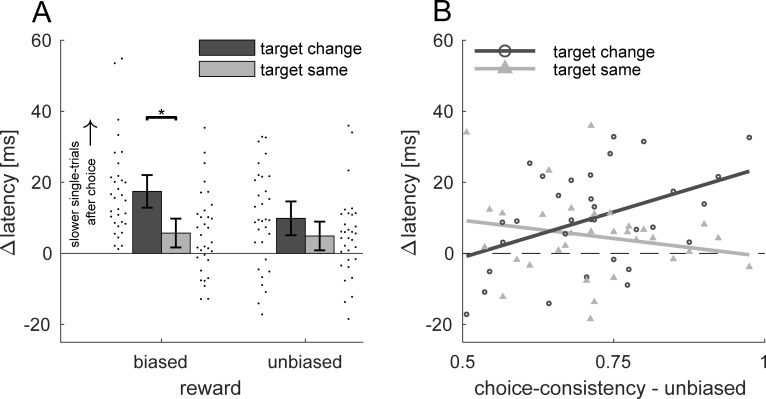
Inter-trial effects. (A) Latency differences between choice-single and single-single sequences for the four different sequence types. Error bars denote 95% confidence intervals of between-participant variability. Black dots next to each bar graph denote values of individual participants in that respective condition. The asterisk indicates a significant difference (p < 0.05). (B) Unbiased inter-trial effects as a function of choice-consistency. Data points denote individual participants, solid lines are linear regressions for the target change (dark) and same (light) condition. Target change and same data of one individual can be linked by the same value of choice consistency on the x-axis.

In a third step, we analyzed inter-trial effects. To this end, we compared single-trial latencies after choice-trials with single-trial latencies after single-trials. Thus, values above 0 indicate that single-trials are slowed down after a choice-trial, whereas values below 0 would indicate that single-trials are faster after a choice-trial. We split up choice-single and single-single sequences into the four distinct sequence types as indicated in Fig 1B. In the biased condition, we expect larger inter-trial effects when the target changes from a choice to single-trial compared to when the target remains the same. This would be a replication of our previous study [17]. If choices exert a retroactive influence, we expect to also find larger inter-trial effects in the unbiased condition if the target changes compared to when it stays the same. In the proactive case, the difference between target change and same in the unbiased condition should be reduced or even completely absent. Inter-trial effects in all conditions (biased/target-change: 17.5 ms, biased/target-same: 5.72 ms, unbiased/target-change: 9.86 ms, unbiased/target-same. 4.92 ms) were significantly above zero (Fig 4A), indicating that single-trial responses were generally slower after a choice-trial (all *t*(31) > 2.3, *p* < 0.024, *d* > 0.4). We compared inter-trial effects using a 2×2 repeated-measures ANOVA with the factors reward-bias (biased versus unbiased) and target (target change versus target same). The ANOVA revealed no main effect of reward-bias, *F*(1,31) = 3.88, *p* = 0.058, *η*_*p*_^2^ = 0.111, but a main effect of target, *F*(1,31) = 9.67, *p* = 0.004, *η*_*p*_^2^ = 0.238. On average, inter-trial effects were larger after a target change (M = 13.7 ms) compared to when the target remained the same (M = 5.3 ms). The same is true for the biased condition, inter-trial effects were larger after a target change compared to when the target remained the same, *t*(31) = 4.2, *p* < 0.001, *d* = 0.74. However, this was not true for the unbiased condition, *t*(31) = 1.36, *p* = 0.183, *d* = 0.24, *BF*_*10*_ = 0.438. Yet, the target × reward-bias interaction was not significant, *F*(1,31) = 3.5, *p* = 0.071, *η*_*p*_^2^ = 0.102.

Given the wide range of choice-consistencies (0.51–0.97) and the ambiguity of inter-trial effects in the unbiased condition, we explored whether the strength of inter-trial effects is related to the choice consistency in the unbiased condition (Fig 4B). We did not perform this analysis for the biased condition because of the limited range of choice-consistencies (Fig 2A). In sequences where the target remained the same, we observed no correlation between choice-consistency and choice-induced inter-trial effects in the unbiased condition, *r*(30) = -0.2, *p* = 0.266, *BF*_*10*_ = 0.4. When the target changed, however, we observed a positive correlation between choice-consistency and inter-trial effects, *r*(30) = 0.432, *p* = 0.014. Thus, the more consistent the idiosyncratic choice behavior in the unbiased condition, the more subsequent responses to the non-chosen target were slowed down. Interestingly, the regression predicted an absence of inter-trial effects when choices were maximally inconsistent (i.e. 50%). This suggests that inter-trial effects only occur when choices are biased.

## Discussion

We made use of inter-trial effects to investigate how non-selected options are suppressed during decision-making. Specifically, we wanted to know whether the suppression of alternative motor plans arises from proactive preparation for upcoming decisions or from retroactive influence from previous decisions. After choice-trials, single-trial responses were generally slower, suggesting that choice-trials trigger cognitive control due to conflict monitoring [33]. In blocks where choices were biased by a difference in reward, single-trial responses to the less-preferred target were slower, replicating previous studies [17,19,20]. When choice-trials were unbiased, the strength of inter-trial effects for changing targets was modulated by the consistency in choice behavior of individual participants. Inter-trial effects decreased in strength the more choices were balanced (Fig 4B). If the alternative action plans were automatically suppressed after motor execution (retroactive), then non-selected action plans should be inhibited in the subsequent trial, no matter whether there was an external reason to prefer one target over the other or not. In this case, we thus would have expected to find inter-trial effects of a similar magnitude in the biased and unbiased condition. However, the present data suggests that the suppression of alternative action plans is not strictly coupled to motor execution of individual action plans. Rather, the relationship between choice preference and the strength of inter-trial effects for previously non-selected actions (target change) in the unbiased condition, suggest that these inter-trial effects arise as a consequence of the extrinsic or intrinsic preference for one target over the other.

In our study, participants could either decide between two targets of equal (unbiased) or between two targets differing in reward (biased). We made sure that our instructions did not prescribe a certain choice behavior, neither for biased nor for unbiased choices. Whereas reward differences successfully biased choice behavior, choice consistency in the unbiased condition was highly variable. It is possible that this behavior depended on the individual strategy with some participants aiming at balanced or random choice behavior [34] while others strategically selected one target they would prefer throughout a block. However, answers given by participants during debriefing suggested that unbiased choice behavior are most likely not explained by conscious strategies: When asked for specific strategies during choice-trials, only one participant noted he/she tried to choose both targets equally often, and two stated they always aimed to select the same choice target. However, answers given during debriefing might not validly represent participants’ behavior. Recent work showed that selection in free-choice paradigms is based on target location rather than target identity [35]. This is consistent with our finding that the majority of participants preferred the same target location across the two unbiased blocks. However, in our task, targets were only defined by their location and could not be identified by any other visual identity (e.g. by their color). Still, choice behavior in the unbiased cannot fully be explained by target location as choice-consistency was significantly reduced compared to the biased condition (Fig 2B).

We observed higher latencies in single-trials when the target appeared at the less preferred location. These biases might build up over the course of the experiment and might reflect the long-term selection history [1,2]. Selection history effects could theoretically explain why latency differences are larger in the biased condition, because here the high reward target is selected more frequently. However, in that case, we should have observed increasing latency differences over the time course of one block. We found no evidence for a temporal modulation of inter-trial effects (S1 and S2 Figs), suggesting that suppression [36] depends more strongly on top-down preferences than on selection history. However, studies reporting selection history effects [6,7] often employ long training phases that contain more trials than our present experiment.

Our task shares some similarities with the negative priming paradigm [9,11,37,38]: If distractors become targets for subsequent responses, response times and accuracy are impaired. Traditionally, negative priming was observed when the identity of the distractor and target were exchanged, yet negative priming can also be observed for spatial selection [39,40]. Although there are obvious similarities in trial sequence and results, we believe our paradigm is not identical to negative priming, because here all stimuli are rewarded targets of which none is externally marked as distractor. Even if one considers the low reward choice target a distractor, this cannot be true for the unbiased condition where both targets were associated with the same reward. Furthermore, paradigms leading to negative priming can also facilitate reaction times (repetition priming) when the target is repeatedly presented [41], which we did not observe in our data. Nevertheless, they might share the same mechanism. For negative priming, episodic retrieval [41,42] or retrieval of stimulus-response associations [25] have been suggested as a source. Our results seem to be inconsistent with these retrieval mechanisms. If our inter-trial effects were caused by stimulus-response retrieval, we would have expected (i) inter-trial effects of the same magnitude for a target change in the biased and unbiased condition and (ii) that inter-trial effects in the unbiased condition were not modulated by choice consistency. Whereas we found no clear evidence, but only a tendency that inter-trial effects associated with a target change are higher in the biased condition, the relationship between choice consistency and the strength of inter-trial effects seems to be at odds with the idea of stimulus-response retrieval. Alternatively, this finding might suggest that the possible encoding of task, context of stimuli into a common event file [23] are modulated by top-down signals.

Whereas negative priming is traditionally described in cognitive tasks, inter-trial effects have also been observed for motor sequences. For saccade eye movements, several previous trial effects have been reported [10]: Saccades latencies are decreased when the same saccade vector is repeated [43] and increased when the previous saccade vector is inverted [44,45] and the previous fixation location is thus refixated (inhibition of return). If one considers the refixation in-between two trials, then both, vector repetition and inhibition of return, would predict higher latencies for single-trials to targets that have been chosen beforehand. This is not the case in our present and previous results [17]. Moreover, the inter-trial effects reported here cannot be explained in terms of inhibition of return or repeated saccade vectors, because of the way inter-trial effects were computed. We computed inter-trial effects as the latency difference between choice-single and the corresponding single-single sequence. As both sequence types contain the same order of saccade vectors, inter-trial effects cannot be explained by the saccade vector sequence but must be related to the decision itself.

Previous studies showed that the magnitude of inter-trial priming of locations can be modulated by the amount of reward [15,46]. In light of these findings, differences in inter-trial effects between the biased and unbiased condition could be attributed to a simple reward modulation if one considers these conditions as high and low reward conditions. However, we think that our results cannot be explained by a simple modulation by reward because of three reasons: First, our previous study [17] reported inter-trial effects that occurred specifically after choice-trials but not after single-trials, although reward was biased in that study for both, choice- and single-trials. A simple modulation by reward would predict latency differences for targets on the same or the other side after single-trials as well. Hence, we were not looking at a general modulation by reward, but an effect specifically related to choices. Second, this choice-related effect in our previous study [17] was the same no matter whether the reward difference between the two choice targets was small or large. Third, in our current study, the dependency of inter-trial effects on choice-consistency in the unbiased condition cannot be explained by a reward modulation. A simple modulation by reward would predict small (because of low reward), but constant inter-trial effects, independent of choice-consistency.

We believe that our results have implications for our understanding how different signals interact in the computation of priority for visual and oculomotor selection. Visual and oculomotor selection are typically explained in terms of a priority map [47–51]. A priority map combines the physical salience of a visual scene with behavioral goals and relevance of objects or regions within the scene. The map location with the highest activity is then selected in a winner-takes all mechanism. Neural correlates of a priority map have been found in the lateral intraparietal area [52], the intermediate layers of the superior colliculus [53] or the frontal eye fields [54] and it was recently suggested that these three areas work together as a global priority map [51]. Recent work emphasizes that selection history in addition to bottom-up and top-down signals act upon the priority map [2,4,47]. History-driven selection can take place at longer timescales, for example when a test phase is preceded by a reward learning phase [6,7] and at short timescales, for example in inter-trial priming [15,55]. Recent EEG results [19] argue against the notion that inter-trial priming arises from lingering inhibition of the previous trial. In contrast, they suggest that inhibition builds up at the beginning of a trial as a consequence of the top-down preference. The current study suggests that inter-trial priming is strongest when participants show a strong preference of one target over the other, thus when a strong top-down signal is present.

## Supporting information

S1 Fig Single-trial latencies from one exemplary participant split according to previous trial type (single versus choice), reward type (biased versus unbiased) and whether the transition from the last to the current trial implied a change in target location or not (change versus same). Black dots denote data from individual trials. Solid black lines are regressions of saccade latencies on the trial number within that block.
(PNG)

S2 Fig Aggregated slope values of the conditions depicted in S1 Fig. Error bars denote 95% confidence intervals across individuals.
(PNG)
