# Supplementary figures and images for: Choice-induced inter-trial inhibition is modulated by idiosyncratic choice-consistency
Source: PLoS One. 2019 Dec 26;14(12):e0226982. doi: 10.1371/journal.pone.0226982 (PMC6932778; doi:10.1371/journal.pone.0226982)

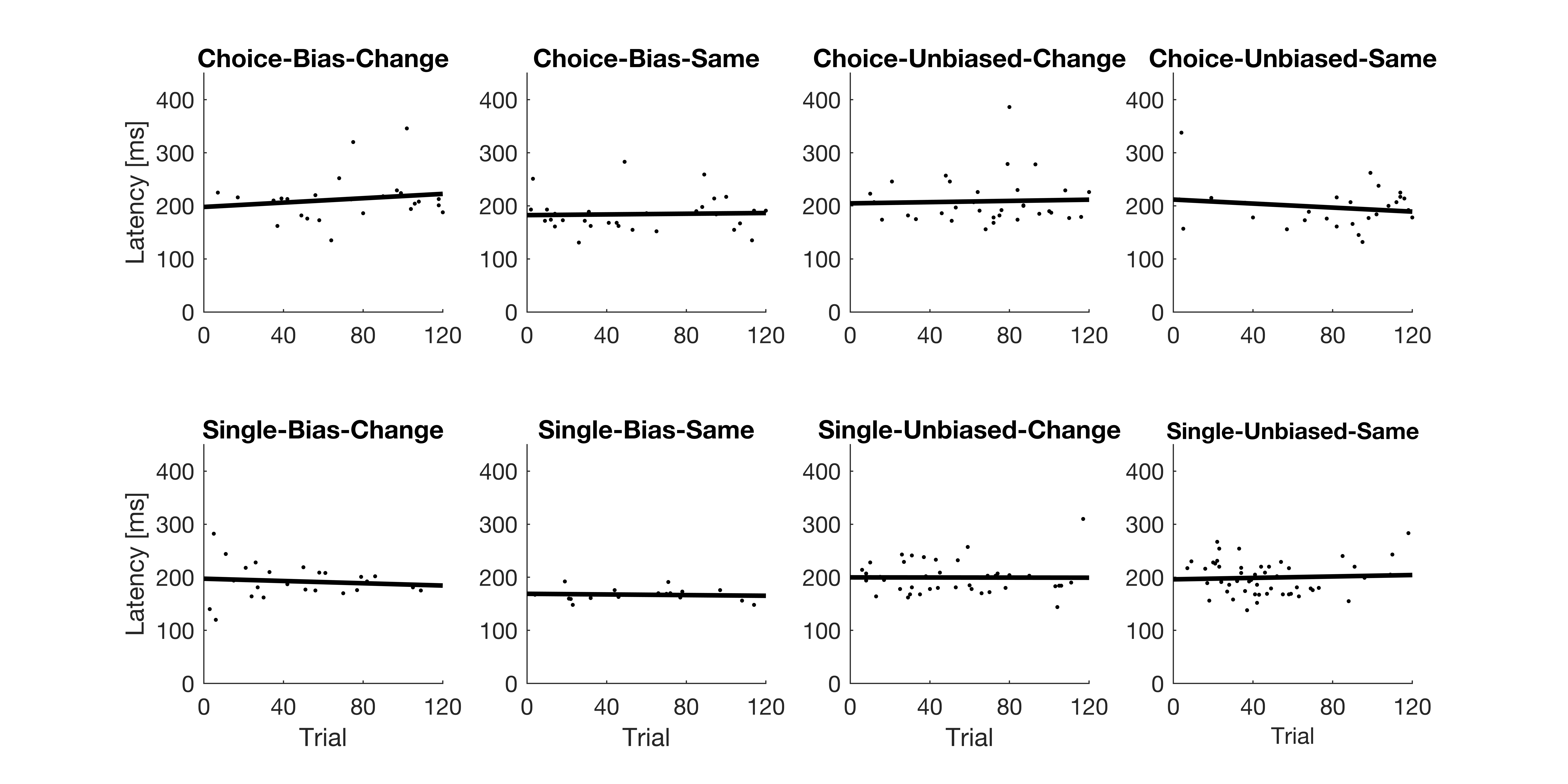

Supplement: S1 Fig — Black dots denote data from individual trials. Solid black lines are regressions of saccade latencies on the trial number within that block. (PNG) [file pone.0226982.s001.png]

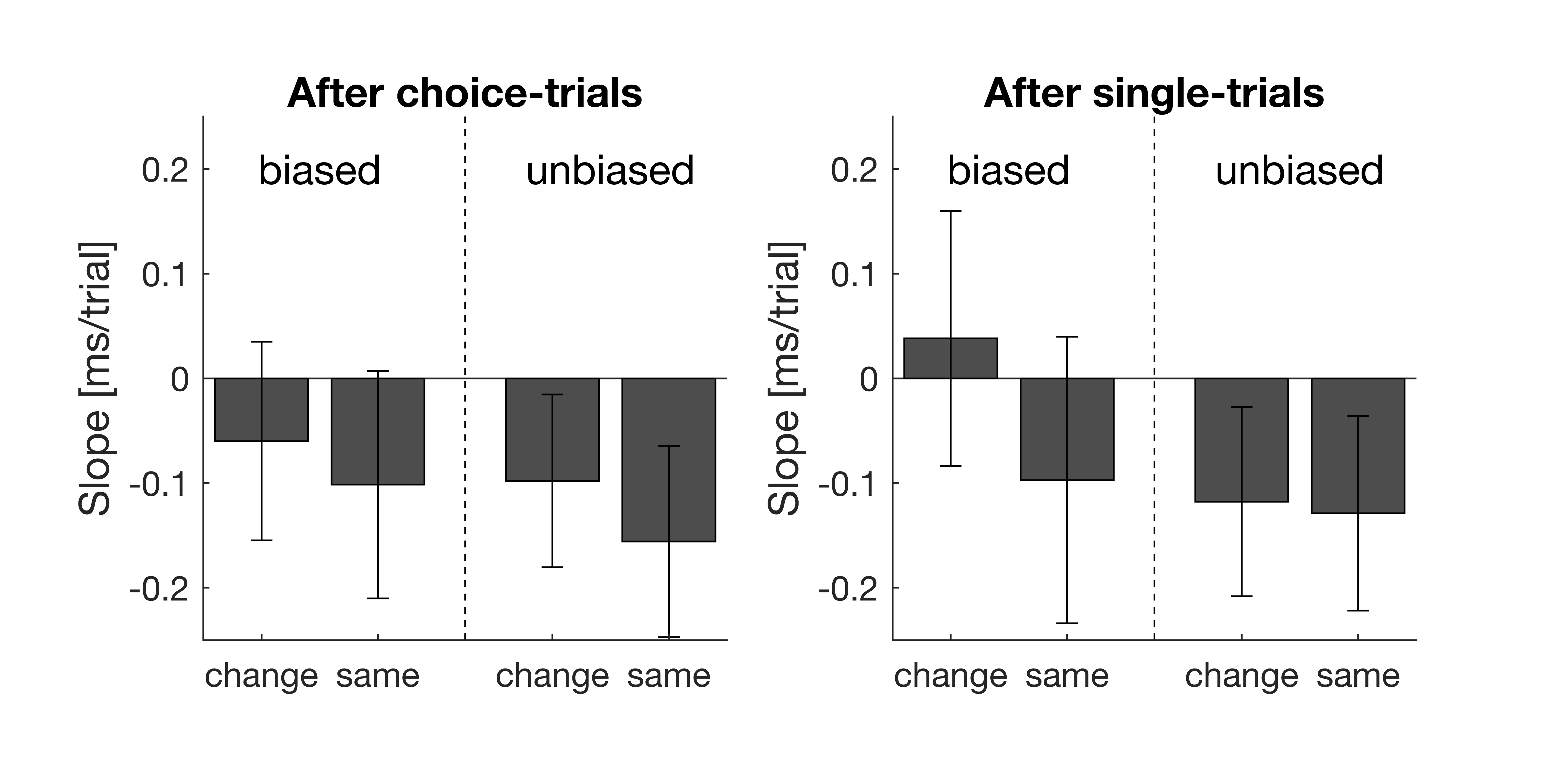

Supplement: S2 Fig — Error bars denote 95% confidence intervals across individuals. (PNG) [file pone.0226982.s002.png]
